# Breast Cancer Screening in Asian Women with Dense Breast by Mammography: A Cross-Sectional Observational Study

**DOI:** 10.31557/APJCP.2021.22.4.1165

**Published:** 2021-04

**Authors:** Jung Sun Lee, Minkyung Oh

**Affiliations:** 1 *Department of Surgery, Haeundae Paik Hospital, College of Medicine, Inje University, Busan, Korea. *; 2 *Department of Pharmacology, Inje University College of medicine, Clinical Trial Center, Inje University Busan Paik Hospital, Busan, Korea. *

**Keywords:** Breast, density, interval cancer, mammography, screening

## Abstract

**Objective:**

Mammography density of Asian women is known to be higher than Western women. After 2009, the Korean National Cancer Screening Program (NCSP) has started to notify mammography density (MD). To investigate the effect of MD notification, we integrated screening results with national health insurance claim data from 2009 to 2013.

**Patients and Methods:**

We performed a cross-sectional observational study which investigated the crude detection rate (CDR), positive predictive value (PPV), and incidence rate of Interval Cancer (IC). IC was defined as breast cancer, where the interval between the screening date and date of diagnosis was more than 12 months and less than 24 months among participants with previous normal results.

**Results:**

CDR and PPV per 100,000 results increased from 510.9 to 756.2 and from 1842.5 to 3364.9, respectively. The incidence rate of IC per 100,000 negative results increased from 623.3 to 676.2. Women younger than 50 years had a high incidence of ICs.

**Conclusion:**

After notifying MD, the incidence rate of IC less increased comparing with CDR or PPV. Screening mammography could be more useful to Asian women when reporting MD.

## Introduction

Mammographic screening is one of many screening tests available worldwide. It is used in the early detection of breast cancer, resulting in less intensive treatment and a better overall prognosis. Thus, the effectiveness of mammographic screening is based on its ability to detect of breast cancer earlier, when the lesion is small, thereby interrupting the natural history of the disease. 

In the past two decades, widespread mammographic screening and effective treatment modalities have led to a shift in the tumor stage at presentation, thereby reducing mortality (Benson et al., 2009; Gotzsche et al., 2013; Nelson et al., 2016). At least 9 separate randomized clinical trials, comprising more than 600,000 women, were conducted in Canada, the United States, the United Kingdom, Russia, and Sweden. All of them showed reduction in breast cancer-related mortality using mammographic screening (Nelson et al., 2016).

Despite the high sensitivity reported for population-based screening programs (ranging from 74.7–89.4%), a percentage of tumors remain undetected, manifesting clinically between a normal screening result and the following screening (Tornberg et al., 2005). These tumors, called interval cancers (IC), are a major limitation of screening programs. The detection rate of IC has been recognized as a valid indicator of both screening quality, and the sensitivity of the screening procedure (Lekanidi et al., 2017; Loy et al., 2015). The number of published studies that report IC on a national level is scarce (Lee et al., 2015; Hofvind et al.,2009; Bennett et al., 2011).

Several previous studies have confirmed the importance of breast density, both in predicting overall breast cancer occurrence, and reducing mammography sensitivity leading to a higher proportion of ICs diagnosed (Lowery et al., 2011; Kerlikowske et al., 2015; Choi et al., 2016). In Korea, the incidence rate of IC among participants with known mammographic density has been previously examined. However, a previous report used a screening interval of 12 months, while the real interval is 24 months(Lee et al., 2016). The aim of this study was to survey the effect of MD notification on the crude detection rate (CDR), positive predictive value (PPV), and incidence rate of IC, using the actual screening interval (24 months), in South Korea.

## Materials and Methods

*Study population*

The National Cancer Screening Program (NCSP) recommends that all Korean women older than 40 years participate in biennial mammographic screening. The baseline cohort comprised 9,469,234 women aged 40 years and older who underwent screening via the NCSP between 2009 and 2013. Among them, 5,907 subjects with a previous diagnosis of breast cancer were excluded, as were 6,051,915 subjects with unknown mammographic breast density. Therefore, a total of 3,417,319 Korean women aged 40 years and older were enrolled in a final analysis. The year of examination was determined by the year of birth; women with an odd number birth year underwent biennial screening on odd number years. 

*Ethical Approvals*

The current study collected data from the NCSP database, which included information on the participant’s demographic characteristics and screening results. Written informed consent was received from participants for the collection of their screening results. Permission was granted by the Ministry of Health and Welfare, and the investigators used data maintained and de-identified by the National Health Insurance Sharing Service (NHIS). We collected data regularly from the NHIS and the need for informed consent for this specific study was waived because the NCSP database is quite large and unidentical personal dataset was released for public health or academic study from NHIS ( NHIS-2018-1-211). Additionally, the study was approved by the Institutional Review Board (IRB) of the Inje University Haeundae-paik hospital, Korea (IRB no. 2017-07-639-001). 

*Measurement*

*Selection and processing of variables*

Participant identification (ID), age, screening date, and screening results were selected from the NCSP breast screening data, and disease codes were selected from the health insurance claim data. Participants were classified according to age at the time of screening as 40–49, 50–59, 60–69, and <70 years, and the screening results were reported as normal, benign disease, suspected breast cancer, or deferred. According to the guidelines on reporting results and recommendations of the Korean NCSP, deferred cases are those in which a judgment of normal, benign lesions, or suspected cancer cannot be made based only on the current examination, and additional examination, such as ultrasound or magnified views, compared with previous mammograms, or re-examination after some period is recommended for accurate diagnosis. Using the disease codes in the health insurance claim data, breast cancers were classified as either malignant neoplasm or carcinoma in situ. The date of breast cancer detection was defined as the first visit day (hospitalization or outpatient) to a medical institution for chemotherapy or surgery. That is, participants were defined as having breast cancer if they were diagnosed with malignant neoplasm or carcinoma in situ, and received either medication or surgery related to breast cancer. Screening data and insurance claim data on breast cancer were merged according to the subjects’ ID.

*Key breast cancer screening performance indicators and their definitions*

From the results of breast cancer screening, suspected and deferred breast cancer diagnoses were defined as positive screening outcomes, while results showing normal findings and benign lesions were defined as negative. Among the participants receiving breast cancer screening, breast cancer treatment occurring within 6 months of screening was defined as breast cancer detected by screening, while the absence of treatment for breast cancer within the period was defined as non-detection.

*Crude detection rate (CDR) for breast cancer screening*

The crude detection rate for breast cancer screening was defined as the number of participants with positive results in whom breast cancer was detected per 100,000 breast cancer screening participants

*Positive predictive value (PPV) of breast cancer screening*

The PPV of screening for breast cancer was defined as the number of detected patients with breast cancer per 100,000 who received positive results on breast cancer screening.

*Interval cancer rate (ICR)*

IC was defined as a breast cancer diagnosis within the interval between screening dates of more than 12 months and less than 24 months in participants with previous negative screening results. ICR was defined as the number cases of breast cancer detected among 100,000 participants with negative breast cancer screening results.

*Statistical analysis*

Data analyses were performed using SAS statistical software (SAS Institute, Cary, NC, USA). The study subjects were selected using breast cancer screening, and health insurance claim data as described above. The variables were presented as counts and percentages, and the PPV, and incidence of IC were calculated. 

## Results

*CDR of breast cancer screening after notification of mammography density*

**Figure 1:**
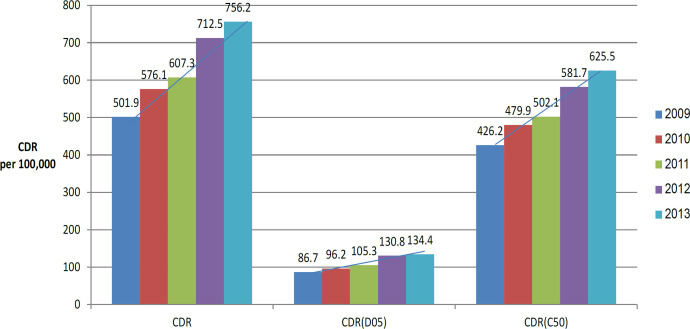
Annual Trends of CDR from 2009 to 2013. CDR per 100,000 participants was increasing from 2009 to 2013. Trends of CDR of in situ cancer and of invasive cancer were also similar. Abbreviations: CDR, crude detection rate; PPV, positive predictive value; D05, in situ cancer; C50, Invasive cancer; CDR, the number of patients who tested positive and were detected with breast cancer / number of screening participants × 100,000

The CDR of positive breast cancer screening increased from 510.9 per 100,000 in 2009, to 756.2 per 100,000 participants in 2013 ( Figure 1). During the same period, the CDR of both carcinoma in situ and invasive cancer also increased. In this 5 year period, the proportion of carcinoma in situ and of invasive cancer decreased among women in their 40s, but increased in those older than 70 years (Supplementary 1).

*PPV of screening for breast cancer after notification of mammography density*

**Figure 2:**
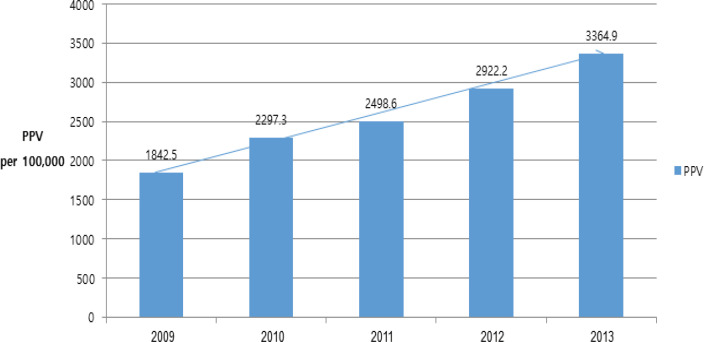
Trends of PPV from 2009 to 2012. Abbreviations: PPV, positive predictive value; PPV, number of breast cancer cases / number of cases with positive screening results × 100,000

The PPV of positive breast cancer screening rapidly increased from 1,842.5 per 100,000 in 2009, to 3,364.9 per 100,000 in 2014 ( Figure 2). The proportion of both in situ, and invasive carcinoma according to positive screening results are different. Cancer detection rate was higher in deferred groups than groups with suspected breast cancer, and the proportion was increasing from 2009 to 2012 (Supplementary 2), although cancer detection rate among suspected breast cancer was decreasing. 

*Incidence rate of IC in breast cancer after notification of mammography density*

**Figure 3:**
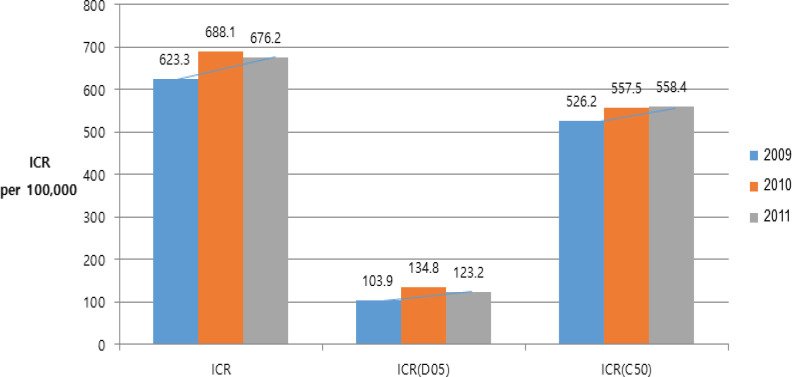
Trends of ICR from 2009 to 2013. ICR per 100,000 participants was increasing from 2009 to 2013. Both ICR of in situ cancer and ICR of invasive cancer were also increasing. The incidence rate of IC relatively showed a moderate increase comparing with a rapid increase of CDR or PPV, especially in invasive cancer. Abbreviations: D05, in situ cancer; C50, Invasive cancer IC, interval cancer; ICR, interval cancer rate ICR, number of breast cancer cases detected / number of cases with negative screening results from 24 months ago × 100,000

The incidence rate of IC among negative participants increased from 623.3 per 100,000 in 2009, to 676.2 per 100,000 participants in 2011 and the incidence rate of both interval carcinoma in situ, and invasive cancer increased (Figure 3) but showed a moderate increased comparing with CDR. The incidence rates of interval carcinoma in situ even increased in all age groups, while the incident rate of interval invasive carcinoma decreased among women in their 40s and 60s, and increased among women in their 50s and 70s. During the 5 year period, the incidence rates of both interval carcinoma in situ, and interval invasive carcinoma were highest among women in their 40s (Supplementary 3). 

## Discussion

When considered with MD, the incidence rate of IC was relatively less increasing comparing with CDR from 2009– 2013. Furthermore, this finding is consistent regardless of tumor type (invasive cancer and carcinoma in situ). The ICR among those younger than 50 years was the highest of all the groups.

IC is an important indicator of the quality of a breast cancer screening program and a predictor for its success in reducing breast cancer mortality (Day et al.,1995). According to risk of IC, several studies showed a few risk factors for IC, such as MD (Hofvind et al., 2009; Mandelson et al., 2000), age (Crane et al., 2002; Klemi et al., 1997), screening method (i.e. between film mammography and digital mammography) (Lowery et al., 2011; Kerlikowske et al., 2015), and screening interval (Tornberg et al., 2005; Hofvind et al., 2009), and the their confounding effect.

Sankatsingb et al., (2018) found that the rate of IC at initial screening among women aged 49–51 years was significantly lower for digital mammography than for serial film mammography. This finding corresponds to the results of the DMIST trial, which showed a higher diagnostic accuracy for digital mammography than film mammography or in pre-, and peri-menopausal women with dense breasts, under the age of 50 (Pisano et al., 2008). The difference in the prevalence of IC could be dependent on the year of screening. O’Brien, et al., (2015) showed that in the first year after screening, the interval cancer rate was 5.8 per 10,000 screens, which increased to 13.2 per 10,000 screens in the second year after screening. 

In previous Korean study using other NCSP data, the results of breast cancer screening was inconsistent dependent on either both screening interval or MD measurement. Lee et al., (2016) reported that the incidence rate of IC was 51.7–76.3 per 100,000 negative patients in which they used 12 months- interval. The CDR of invasive cancer in the present study was higher than that of National Cancer Report in the same period (Ministry of Health and Welfare et al., 2018), because the latter study included all participants regardless of MD reporting.

In contrast, there were no significant differences in the CDR of breast cancer screening between the month before, and the month after the implementation of breast density notification legislation, on breast density reporting by radiologists in United States (Bahl et al., 2016). 

Asian women, including Korean women, characteristically have higher-density breasts than women from other ethnic groups (Dai et al., 2014; Bae et al., 2014; Ohuchi et al., 2016). Consequently, high accuracy is difficult to achieve with mammography screening alone. In addition, the age-specific incidence of female breast cancer in Asia peaks at 40–49 years, whereas in Western countries, the peak is at 60–70 years (Leong et al., 2010). Asian countries must, therefore, take measures to address the accuracy of breast cancer screening in women aged 40–49 years (Ohuchi et al., 2016). 

According to the Japan Strategic Anti-cancer Randomized Trial (J-START) to investigate the efficacy of adjunctive ultrasonography, it reported that interval cancers were detected in the ultrasonography plus mammography (0.05%) group, compared with the mammography-only group (0.10%). Additional ultrasonography could reduce interval cancer among women aged 40–49 years (Ohuchi et al., 2016). Other investigators (Berg et al., 2008; Corsetti et al., 2011; Corsetti et al., 2008) had previously shown low interval cancer rates among women screened with adjunctive ultrasonography. 

Ongoing controversy over the optimal approach to breast cancer screening has led to discordant professional society recommendations, particularly for women aged 40–50 years. One potential solution, risk-based screening, has been on the rise, where decisions around the starting age, stopping age, frequency, and modality of screening are based on individual risk, so as to maximize the early detection of aggressive cancers, and minimize the harms of screening through optimal resource utilization (Shieh et al., 2017; Kerlikowske et al., 2019). 

This study had a number of limitations. First, as health insurance claim data were used to detect breast cancer in the central cancer registry, there may have been some differences between the actual incidence of breast cancer in the Central Cancer Registry, and the cases detected in the present study. In fact, the data from the present study excluded patients with end stage breast cancer receiving only palliative treatment with no anticancer treatment or surgery. However, the number of detected breast cancer ranged from 97.3% to 101.1% of the values from the central cancer registry during 2009–2012. In addition, as most patients with cancer qualified for special case calculation, the difference between the number of the detected breast cancers in this study and the number in the Central Cancer Registry was unlikely to be large enough to significantly influence our results. Second, the detection of breast cancer may be influenced by symptoms, level of exposure to risk factors such as pregnancy, breast feeding, hormone treatment, and family history of breast cancer, socioeconomic, and educational level. These factors could influence either the breast density, or behaviors after notified a screening result. We did not include these personal factors.

Participants were not well-informed on interval cancer, and the limitations of mammography, because the rate of IC was relatively low in the biennial screening interval. Being in a specific age- group, or having a strong family history, in addition to reported mammographic density could assist clinicians with choosing supplemental examination. The present study showed the specific group who needs attention by both clinicians, and policy-makers. 

In conclusion, both CDR, and incidence of IC have increased during the 5 year period (2009–2013). After reporting MD, the incidence rate of IC relatively less increased comparing with CDR or PPV. Screening mammography was probably considered to be useful in Asian women when reporting MD. Though women whose last screening results were normal or benign disease, participants should be announced to take care of their breast health considering other risk factors, including age, and MD. 

## Author Contribution Statement

Conceptualization: J.Lee. Data curation: M.Oh. Formal analysis: M.Oh. Methodology: J.Lee. Software: M.Oh. Investigation: M.Oh, J.Lee. Writing- original draft: J.Lee. Writing-review & editing: J.Lee, M.Oh

## References

[B1] Bae JM, Shin SY, Kim EH (2014). Distribution of dense breasts using screening mammography in Korean women: a retrospective observational study. Epidemiol Health.

[B2] Bahl M, Baker JA, Bhargavan-Chatfield M (2016). Impact of breast density Notification legislation on Radiologists’ practices of reporting breast density: A Multi-State study. Radiology.

[B3] Bennett RL, Sellars SJ, Moss SM (2011). Interval cancer in the NHS breast cancer screening programme in England, Wales and Northern Ireland. Br J Cancer.

[B4] Benson JR, Jatoi I, Keisch M (2009). Early breast cancer. Lancet.

[B5] Berg WA, Blume JD, Cormack JB (2008). Combined screening with ultrasound and mammography vs mammography alone in women at elevated risk of breast cancer. JAMA.

[B6] Choi WJ, Cha JH, Kim HH (2016). Analysis of prior mammography with negative result in women with interval breast cancer. Breast Cancer.

[B7] Corsetti V, Houssami N, Ferrari A (2008). Breast screening with ultrasound in women with mammography-negative dense breasts: evidence on incremental cancer detection and false positives, and associated cost. Eur J Cancer.

[B8] Corsetti V, Houssami N, Ghirardi M (2011). Evidence of the effect of adjunct ultrasound screening in women with mammography negative dense breasts: interval breast cancers at 1 year follow-up. Eur J Cancer.

[B9] Crane CE, Luke CG, Rogers JM (2002). An analysis of factors associated with interval as opposed to screen-detected breast cancers, including hormone therapy and mammographic density. Breast J.

[B10] Dai H, Yan Y, Wang P (2014). Distribution of mammographic density and its influential factors among Chinese women. Int J Epidemiol.

[B11] Day N, McCann J, Camilleri-Ferrante C (1995). Monitoring interval cancers in breast screening programmes: the east Anglian experience Quality Assurance Management Group of the East Anglian Breast Screening Programme. J Med Screen.

[B12] Gotzsche PC, Jorgensen KJ (2013). Screening for breast cancer with mammography. Cochrane Database Syst Rev.

[B13] Hofvind S, Yankaskas BC, Bulliard JL (2009). Comparing interval breast cancer rates in Norway and North Carolina: results and challenges. J Med Screen.

[B14] Kerlikowske K, Miglioretti DL, Vachon CM (2019). Discussions of Dense breast, Breast Cancer Risk, and screening choices in 2019. JAMA.

[B15] Kerlikowske K, Zhu W, Tostenson AN (2015). Breast cancer Surveillance Consortium Identifying women with dense breast at high risk for interval cancer. Ann Intern Med.

[B16] Klemi PJ, Toikkanen S, Rasanen O (1997). Mammography screening interval and the frequency of interval cancers in a population-based screening. Br J Cancer.

[B17] Lee K, Kim H, Lee JH (2016). Retrospective observation on contribution and limitations of screening for breast cancer with mammography in Korea: detection rate of breast cancer and incidence rate of interval cancer of the breast. BMC Womens Health.

[B18] Lekanidi K, Dilks P, Suaris T (2017). Breast screening: What can the interval cancer review teach us? Are we perhaps being a bit too hard on ourselves?. Eur J Radiol.

[B19] Leong SP, Shen ZZ, Liu TJ (2010). Is breast cancer the same disease in Asian and Western countries?. World J Surg.

[B20] Lowery JT, Byers T, Hokanson JE (2011). Complementary approaches to assessing risk factors for interval breast cancer. Cancer Cause Control.

[B21] Loy EY, Molinar D, Chow KY (2015). National Breast Cancer Screening Programme, Singapore: Evaluation of participation and performance indicators. J Med Screen.

[B22] Mandelson MT, Oestreicher N, Porter PL (2000). Breast density as a predictor of mammographic detection: comparison of interval and screen-detected cancers. J Natl Cancer Inst.

[B23] Ministry of Health &amp; Welfare Korea Central Cancer Registry, National Cancer Center (2018). Annual report of cancer statistics in Korea in 2012.

[B25] O’Brien KM, Dwane F, Kelleher T (2015). Interval cancer rates in the Irish National Breast cancer screening programme. J Med Screen.

[B26] Ohuchi N, Suzuki A, Sobue T (2016). Sensitivity and specificity of mammography and adjunctive ultrasonography to screen for breast cancer in the Japan Strategic Anti-cancer Randomized Trial (J-START): a randomized controlled trial. Lancet.

[B27] Pisano ED, Hendrick RE, Yaffe MJ (2008). Diagnostic accuracy of digital versus film mammography: exploratory analysis of selected population subgroups in DMIST. Radiology.

[B28] Sankatsingb VDV, Fracheboud J, de Munck L (2018). Detection and interval breast cancer rates during the transition from screen-film to digital mammography in population-based screening. BMC Cancer.

[B29] Shieh Y, Eklund M, Madlensky L (2017). Breast Cancer Screening in the precision medicine era: risk –based screening in a population based trial. J Natl Cancer Inst.

[B30] Tornberg S, Codd M, Rodrigues V (2005). Ascertainment and evaluation of interval cancers in population based mammography screening programmes: a collaborative study in four European centers. J Med Screen.

